# Suspected community-acquisition of carbapenem-resistant hypervirulent Klebsiella pneumoniae (CRhvKp) in Germany: a case report and implications for infection control

**DOI:** 10.3205/dgkh000646

**Published:** 2026-03-20

**Authors:** Nora Helke Leder, Oana Joean, Micha Banz, Claudia Stein, Frank Kipp, Jürgen Rödel, Sabine Trommer

**Affiliations:** 1 Institute for Infectious Diseases and Infection Control, Jena University Hospital, Jena, Germany; 2 Department of Internal Medicine IV (Gastroenterology, Hepatology and Infectious Diseases), Jena University Hospital, Germany; 3 Institute of Medical Microbiology, Jena University Hospital, Friedrich Schiller University, Jena, Germany

**Keywords:** hypervirulent, Klebsiella pneumoniae, infection control, carbapenemase, multidrug-resistant, Gram-negative bacteria

## Abstract

**Aim:**

Hypervirulent *Klebsiella pneumoniae* (hvKp) has emerged as a disease threat due to a higher morbidity and mortality associated with specific virulence genes. The acquisition of carbapenem-resistance (CRhvKp) in some strains limits treatment options, posing a serious clinical challenge. While healthcare-associated transmissions have been reported, the epidemiological dynamics and infection prevention implications of CRhvKp remain insufficiently elucidated. In Germany, routine diagnostics for Gram-negative pathogens rarely include specific identification of hvKp or systematic detection of its virulence markers, and there is no mandatory notification of CRhvKp cases.

**Methods:**

We established a two-step screening approach for the routine diagnostic detection of hvKp, combining loop-mediated isothermal amplification (LAMP) with confimatory whole-genome sequencing (WGS). This workflow was applied to all carbapenemase producing *K*. *pneumoniae* and to clinical isolates considered at increased risk for hvKp.

**Results:**

We report the case of a 1973-born male patient with a history of Child-Pugh class B cirrhosis who was admitted due to acute liver failure and ascites triggered by infection. The patient had no recent travel history but had been recently admitted to local hospitals in Thuringia, Germany. Ascitic fluid obtained by paracentesis appeared putrid and yielded an ESBL-producing and fluoroquinolone-resistant Escherichia coli. In addition, a lower urinary tract infection due to CRhvKp was identified. The CRhvKp infection was deemed community-acquired, with the route of acquisition remaining unknown. The isolate belonged to ST147 and carried the *bla*_NDM-5_ and *bla*_OXA-48_ carbapenemase genes. The patient was successfully treated with cefiderocol (Fetcroja, Shionogi & Co. Ltd.) over the course of 14 days. Standard infection prevention precautions for patients with carbapenem-resistant *Enterobacterales* were applied. No intra-hospital transmission of this strain was detected in our routine WGS-based surveillance.

**Conclusion:**

CRhvKp can occur in patients without establishes epidemiological or clinical risk factors, suggesting a broader reservoir than currently assumed. Further research regarding prevalence, transmissibility, environmental persistence, and colonization dynamics of CRhvKp strains are urgently needed to determine their implications for infection prevention and control in hospitals in Germany.

## Zusammenfassung

**Ziel:**

Hypervirulente *Klebsiella pneumoniae* (hvKp) unterscheiden sich von klassischen *Klebsiella pneumoniae*-Stämmen durch Virulenzdeterminanten, die schwere invasive Infektionen, auch bei zuvor gesunden Personen, begünstigen. Das zusätzliche Vorliegen einer Carbapenemresistenz (CRhvKp) bei einem Teil dieser Stämme schränkt therapeutische Optionen weiter ein und stellt eine erhebliche klinische Herausforderung im Management der Fälle dar. Nosokomiale Übertragungen von CRhvKp wurden bereits beschrieben, jedoch sind die Übertragungswege sowie die Konsequenzen für das krankenhaushygienische Management bislang unzureichend bekannt. In Deutschland erfolgt in der Routinediagnostik nur selten eine gezielte Identifizierung von hvKp, da die systematische Erfassung entsprechender Virulenzfaktoren in der Regel bisher nicht routinemäßig durchgeführt wird. Außerdem besteht in Deutschland aktuell keine Meldepflicht für hvKp ohne Carbapenemasenachweis.

**Methoden:**

In unserer Klinik etablierten wir einen zweistufigen Screening-Ansatz zur routinediagnostischen Detektion von hvKp. Der Workflow kombiniert eine loop-vermittelte isotherme Amplifikation (LAMP) zum Nachweis Hypervirulenz-assoziierter Gene mit anschließenden Ganzgenomsequenzierung (WGS). Eingeschlossen werden alle Carbapenemase-produzierende *K. pneumoniae* sowie Isolate, bei denen aufgrund ihres Phänotyps oder der klinischen Manifestation ein erhöhtes Risiko für hvKp besteht.

**Ergebnisse:**

Ein 1973 geborener Mann mit bekannter Child-Pugh-B-Leberzirrhose stellte sich mit infektgetriggertem akutem Leberversagen und Aszites vor. Der Patient hatte keine kürzlichen Auslandsreisen unternommen, jedoch mehrfach stationäre Aufenthalte in Thüringer Kliniken. Der bei einer Parazentese gewonnene Aszites erwies sich als eitrig; im Rahmen der mikrobiologischen Diagnostik wurde Escherichia coli mit ESBL- und Fluorchinolonresistenz isoliert. Zusätzlich zeigte die Diagnostik eine Infektion der unteren Harnwege durch eine CRhvKp. Diese Infektion wurde als ambulant erworben eingestuft, der genaue Übertragungsweg des Erregers ließ sich nicht bestimmen. Das Isolat gehörte zum Sequenztyp 147 (ST). Mit Hilfe der Ganzgenomsequenzierung konnten die Resistenzgene *bla*_NDM-5_ und *bla*_OXA-48_ nachgewiesen werden. Der Patient wurde über 14 d mit Cefiderocol (Fetcroja, Shionogi & Co. Ltd.) behandelt. Die für Patienten mit Carbapenem-resistenten *Enterobacterales* vorgesehenen Infektionspräventionsmaßnahmen wurden umgesetzt. In unserer routinemäßigen WGS-basierten Surveillance ergaben sich keine Hinweise auf eine nosokomiale Übertragung dieses Bakterienklons.

**Schlussfolgerung:**

CRhvKp kann bei Patienten ohne epidemiologische oder klinische Risikofaktoren nachgewiesen werden, was auf ein breiteres Reservoir als bisher angenommen hinweist. Weitere Untersuchungen zur Prävalenz, Übertragbarkeit, Umweltpersistenz und Kolonisationsdynamik von CRhvKp-Stämmen sind dringend erforderlich, um ihre Bedeutung für die Krankenhaushygiene und Infektionsprävention in Deutschland fundiert bewerten und nachfolgend erforderliche Präventionsmaßnahmen ableiten zu können.

## Introduction

Hypervirulent *Klebsiella pneumoniae* (hvKp) can cause severe infections in previously healthy individuals, including pyogenic liver abscesses, meningitis, and disseminated infections with metastatic spread. In contrast, classical *Klebsiella pneumoniae* (cKp) predominantly causes healthcare-associated infections in multimorbid patients [1].

Although no universally accepted definition of hvKp exists, it is most commonly characterized by the presence of genes encoding for capsular polysaccharides (*rmpA, rmpA2*), leading to a hypermucoviscous phenotype, and siderophore systems (*iro, iuc, ybt*) that enhance the iron uptake [2], [3], [4]. The Kleborate scoring system estimates the combined effect of possible biomarker parings between the siderophores aerobactin *(Iuc)* and salmochelin *(Iro)*, capsular polysaccharides (*rmpA, rmpA2*) and the genotoxin colibactin (*Clb)* to anticipate the virulence of *K.* *pneumoniae* isolates in a virulence score from 1 to 5. This scoring system takes into account existing evidence and was created to estimate the virulence of any *Klebsiella* isolate regardless of the presence of resistance determinants [5]. Rödel et al. [2] selected *rmpA/A2, iuC, iroC, ybt, clb*, in reference to the Kleborate virulence score to detect hvKp isolates. Wahl et al. [6] suggest clinical manifestation and hypermucoviscious phenotype in combination with a Kleborate score >3 or the presence of iuC, ybt and rmpA or rmpA2 as definition for hvKp. Russo et al. [7] proposed a set of biomarkers for *K. pneumoniae* with acquired resistance, comprising of *iucA, rmpA, rmpA2, peg-344* and *iroB*, which are linked to the canonical pLVPK virulence plasmid. 

HvKp strains were first identified in the Asian and Pacific region [4] and have since been reported worldwide [8]. In endemic areas like China, hvKp might replace cKp as dominant pathogen in hospital settings [9]. Globally, sequence type (ST) 23, ST65, and ST86 are among the most frequently detected hvKp lineages [10]. Although hvKp infections remain less frequently reported in Europe than in endemic regions, sporadic cases and small outbreaks suggest that its true prevalence may be underestimated, partly due to limited diagnostic capabilities and awareness.

Hypervirulence and multidrug resistance have historically rarely co-occurred in *K. pneumoniae*. Their combination in carbapenem-resistant hvKp (CRhvKp) is therefore of particular concern, as it unites the ability to cause severe, invasive disease with severely limited treatment options. The gain of carbapenem-resistance in hvKp is often due to the acquisition of plasmids carrying carbapenemase genes [11], [12] . In a rapid risk assessment, the European Centre for Disease Prevention and Control (ECDC) reported an increase in reports of CRhvKp ST23 in Europe and highlighted the importance of timely detection of CRhvKp as well as the importance of infection prevention measures to mitigate further spread [11]. Sequence types described in Europe are ST11, ST147, and ST395, but due to the lack in mandatory reporting the dissemination of these strains in Europe cannot fully be evaluated [8], [10]. In Germany, CRhvKp of ST147, ST395 and ST231 have been documented [13]. Due to the absence of national surveillance data, to date the prevalence of CRhvKp in Germany remains unknown.

The absence of standardized diagnostic methods remains a major factor for the current lack of reliable surveillance data on hvKp [10]. Furthermore, no internationally standardized gene panel exists for hvKp detection; current approaches rely on varying sets of virulence-associated genes proposed by different research groups. The gold standard is to confirm the hypervirulent phenotype in infection models, such as in vivo mouse models, which are reported to have the highest accuracy for hvKp detection [10]. However, these approaches are time-consuming and not feasible in a routine microbiology setting. While a positive string test to assess for hypermucoviscous phenotype is an easy to employ screening tool, the phenomenon is not present across all hvKp isolates, rendering it insufficient as a standalone diagnostic method and therefore not sensitive enough as sole detection method of hvKp [14], [15]. Molecular methods like polymerase chain reaction (PCR) and loop-mediated isothermal amplification-based methods (LAMP) enable the detection of predefined virulence genes [10], but they are inherently limited to the selected genetic targets. In contrast, whole genome sequencing (WGS) provides a comprehensive, target-independent analysis of virulence and resistance determinants, supports retrospective genomic investigation, and allows assessment of transmission events, which offers valuable insights from an infection prevention perspective [16].

Reports of healthcare associated transmissions of both hvKp [9] and CRhvKp [17] underline the need to better understand their transmission dynamics in healthcare settings. Whether the presence of virulence determinants in hvKp or CR-hvKp increases their potential for patient-to-patient spread remains unclear. Notably, a recent study identified disinfectant resistance genes in hvKp isolates [18], raising additional concerns regarding their persistence in the healthcare environment. Despite these observations, the infection prevention and control implications of CRhvKp remain insufficiently elucidated [19].

Neither current guidelines for carbapenemase-producing Enterobacterales (CPE) nor evidence-based recommendations address the specific infection prevention needs of hvKp or CRhvKp, leaving institutions to adapt infection prevention strategies on a case-by-case basis. Here, we report a case of CRhvKp identified in a patient without classical risk factors, illustrating the diagnostic challenges and infection prevention considerations associated with this emerging pathogen.

## Case description

We report the case of a 1973-born male who was referred to our tertiary care university hospital with decompensated alcoholic liver cirrhosis and recurrent massive ascites. His past medical history included chronic pancreatitis, arterial hypertension, and diabetes mellitus. In the months preceding the current admission, the patient underwent multiple hospitalizations in various facilities across Thuringia, Germany, due to recurrent cirrhotic decompensations. In one of these admissions, a rectal colonization and subsequent bloodstream infection with meropenem-resistant *K. pneumoniae* as well as a rectal colonization with extended-spectrum β-lactamase (ESBL)-producing *Escherichia (E.) coli* was reported. The patient was admitted to our hospital before, but in previous admissions, no multi-drug-resistant organisms (MDRO) were detected and the patient did not have contact to patients with known carriage of MDRO. The patient hadn’t travelled outside Germany in the past years. 

On admission, he presented with abdominal pain, nausea, fatigue, and dysuria. Abdominal ultrasonography and computed tomography revealed septated ascites; paracentesis yielded purulent from which an ESBL-producing and fluoroquinolone-resistant *E. coli* was isolated. Empiric treatment with meropenem (Hikma Pharmaceuticals, 1 g three times daily) and linezolid (Panpharma, 600 mg twice daily) for five days was initiated, as a hospital-acquired infection was suspected in light of his recent hospitalizations. 

Given the additional suspicion of a urinary tract infection, urine samples revealed a white blood cell (WBC) count of 4,799 per µl. Culturing yielded a carbapenem-resistant *K. pneumoniae* with a count of 100,000 colony-forming units per milliliter. In our hospital, we established a workflow for the detection of hvKp. This workflow included

1. isolates with carbapenemase production,
2. isolates from invasive deep tissue infections, periprosthetic infections, abscesses, meningitis, otitis and ocular infections, as well as
3. isolates exhibiting a distinctive morphology combined with a positive string test.

The two-stepped approach includes a loop-mediated isothermal amplification (LAMP)-based screening for hypervirulence-associated genes* rmpA/A2, iuC, iroC, ybt, clb* using the IVDR-approved eazyplex^®^ hvKp (AmplexDiagnostics, Gars-Bahnhof, Germany) with subsequent whole genome sequencing (WGS) as previously described [20] for genomic surveillance. In addition to hvKp, our genomic surveillance includes all carbapenemase-producing Enterobacterales detected in our hospital.

The *K. pneumoniae* isolate belonged to ST147. Molecular testing confirmed the presence of hypervirulence-associated genes *iucA, iucB, iuC, iucB* and *ybt*. The isolate scored 4 points in the Kleborate Virulence-Score. The strain carried both *bla*_NDM-5_ and *bla*_OXA-48_ carbapenemases genes. Based on these findings, antibiotic therapy was switched to cefiderocol (Fetcroja, Shionogi & Co. Ltd.) for 14 days.

In accordance with national recommendations for the management of carbapenem-resistant organisms [21], the patient was isolated in a single room for the entire hospital stay, with healthcare personal adhering to personal protective equipment protocols, and a thorough terminal disinfecting cleaning protocol for the patient´s room after discharge was conducted. To date, no genomically related isolates were detected during or after his hospitalization in our routine WGS-based molecular surveillance.

## Discussion

Here, we presented the case of CRhvKp-detection in a patient without recent travel to regions where CRhvKp is considered endemic. 

The isolate carried two out of five biomarkers selected for our LAMP-based screening, namely iuC (aerobactin) and ybt (yersiniabactin) [2]. The cooccurrence of aerobactin and yersiniabactin has repeatedly been identified as key constellation in isolates with suspected hypervirulence [5], [6] and corresponds with a high level of suspected virulence in the Kleborate score [5]. Contrarily, rmpA/rmpA2 were not detected. Although these genes are included in several diagnostic panels for hvKp, none of the commonly used frameworks define rmpA/rmpA2 or the associated hypermucoviscousity phenotype as mandatory criteria for hvKp classification [2], [5], [7]. Application of the biomarker set published by Russo predicted a low probability of hypervirulence [7]. These contradicting assessments highlight the ongoing lack of consensus regarding molecular definitions of hvKp. The criteria for hvKp proposed by Wahl et al. [6] advocate for a nuanced expert assessment of isolates and emphasize the combination of molecular biomarkers, phenotypical characteristics and the clinical presentation. Notably, the isolate presented in this case study was recovered from a clinically relevant specimen in the context of infection. The patient also previously experienced a bloodstream infection caused by a carbapenem-resistant *K. pneumoniae*, however, due to the absence of molecular characterization of that isolate, it remains unclear whether both episodes were caused by the same strain or shared virulence or resistance determinants. In evaluation of the detected biomarkers and the clinical presentation, we opted to prioritize the Kleborate score and assessed the isolate as CRhvKp.

The suspected urinary tract infection caused by the CRhvKp was successfully treated with cefiderocol, which is recommended for infections caused by carbapenem-resistant Gram-negative bacteria [22], [23]. However, cases of cefiderocol resistance in CRhvKp have been reported [13]. In a recent *in vitro* study, CRhvKp isolates had a higher siderophore production compared to carbapenem-resistant *K. pneumoniae* lacking hypervirulence traits, potentially reducing cefiderocol susceptibility [24]. Further studies are needed to clarify the effect of siderophore-mediated virulence on the clinical effectiveness of cefiderocol in treating CRhvKp infections. 

While an antibiotic treatment was initialized, asymptomatic bacteriuria in combination with abdominal pain and dysuria caused by the ascites could not be excluded for this patient. Despite the detection of CRhvKp, the indication for treatment has to be evaluated thoroughly to avoid overtreatment for the patient and reduce the selective pressure. From an antimicrobial stewardship and infection prevention perspective, expert clinical consultation is essential to avoid relying solely on scoring systems such as the Kleborate score. Rather, therapeutic decisions should be guided primarily by the patient’s clinical presentation. 

Infection prevention measures were applied due to the isolate’s carbapenem resistance. Further research needs to investigate, if infection prevention measures established for CPOs should also be applied to carbapenem-susceptible hvKp. Environmental sampling of the patient’s room was not performed in this case. However, *K. pneumoniae* can persist on surfaces for several weeks [25] and carbapenem-resistant *K. pneumoniae* from biofilm reservoirs has recently been described as sources of hospital-associated transmission [26]. Further studies need to investigate the role of the hypervirulence-associated genes in persistence, replication capacity and transmissibility of hvKp.

In our case report, the CRhvKp isolate was classified as community-acquired, but the route of acquisition could not be determined and acquisition during previous hospital admissions to another regional hospital cannot be ruled out. The isolate belonged to ST147, which has also recently been reported in Ukraine [27], the UK [28], Italy [29] and Ghana [30]. Given the patient’s history, a recent acquisition from an endemic region appears unlikely. Alternatively, undetected acquisition within the German healthcare system is conceivable. While CRhvKp strains have occasionally been reported in Germany [31], the absence of standardized detection methods and mandatory reporting hampers an accurate assessment of their spread.

With our two-stepped approach, we established a structured workflow within routine diagnostics aimed at systematically identifying a high proportion of hvKp isolates in our hospital. The LAMP-based screening enabled rapid identification of hvKp-associated gene loci to support clinical decisions, while the WGS-based surveillance enabled comprehensive assessment of the virulence and resistance gene repertoire and enables prospective monitoring of the molecular epidemiology and potential intrahospital transmission events. A multistep approach combining string test, PCR with *magA, iutA, rmpA* and *rmpA2* as targets, together with WGS, was previously demonstrated as a viable way to detect hvKp in a study from Neumann et al. [32]. While a PCR-based-screening offers fast detection of the targets, the absence of a universal definition of hvKp-associated genes limits broader implementation. Heiden et al. used WGS to thoroughly characterize virulence genes of CRhvKp in combination with experiments for phenotypic assays such as biofilm formation, hypermucoviscosity, and siderophore secretion in an outbreak setting in a German hospital [33]. Dogan et al. [34] combined WGS with *in* *vivo* infection models to characterize the virulence of hvKp isolates. While such approaches provide comprehensive insights into pathogenicity, they are not feasible for routine diagnostics due to their high workload, complexity, and long turnaround times. 

Non-resistant hvKp strains are not systematically captured through our surveillance program. Instead, our workflow follows a cost- and workload-effective approach by only screening *K. pneumoniae* isolates from invasive deep tissue infections, periprosthetic infections, abscesses, meningitis, otitis and ocular infections, as well as isolates harbouring carbapenemase genes, rather than screening all *K. pneumoniae* isolates for hypervirulence. Consequently, the diagnostic yield of our workflow depends on which *K. pneumoniae* isolates are selected for inclusion in the diagnostic pathway. Our approach offers broader genomic resolution than PCR-based methods and is more feasible for routine diagnostics than in vivo model–based workflows.

## Conclusions

The presented case demonstrates CRhvKp-detection in a patient without travel to endemic regions, suggesting a broader reservoir than currently anticipated. It highlights the diagnostic and infection prevention challenges posed by CRhvKp and illustrates the value of incorporating structured hypervirulence screening and genomic surveillance into routine workflows. Such an approach enables rapid identification in combination with prospective molecular surveillance needed to guide local infection prevention measures in the absence of national or international guidance. Further research on CRhvKp, especially regarding prevalence, transmissibility, environmental persistence, and colonization dynamics of CRhvKp strains are urgently needed to determine the infection prevention implications of CRhvKp in Germany to guide a larger scale public health response.

Given the lack of a universally accepted molecular definition of hvKp and the resulting diagnostic uncertainty, close interdisciplinary collaboration between clinical microbiology, infectious diseases, infection prevention and control, and genomic epidemiology is essential to ensure appropriate interpretation of results and to translate molecular findings into proportionate clinical and infection control actions.

## Notes

### Competing interests

The authors declare that they have no competing interests.

### Authors’ ORCIDs

- Leder NH: https://orcid.org/0009-0005-1465-1853
- Joean O: https://orcid.org/0000-0002-5995-4605
- Banz M: https://orcid.org/0000-0001-8813-5388
- Stein C: https://orcid.org/0000-0002-5899-3485
- Kipp F: https://orcid.org/0009-0006-3360-489X
- Trommer S: https://orcid.org/0009-0007-2482-1398

### Funding

None. 
